# Bilateral exudative retinal detachment with subretinal light-chain protein in a patient with multiple myeloma -case report-

**DOI:** 10.1186/s12886-024-03739-5

**Published:** 2024-10-31

**Authors:** Arved Rikus Gruben, Christoph Ehlken, Johann Roider

**Affiliations:** Department of Ophthalmology, University Hospital Schleswig Holstein, Arnold-Heller Str. 3, 24105 Kiel, Germany

**Keywords:** Retinal detachment, Plerixafor, Stem cell mobilization, Adverse event, Case report

## Abstract

**Background:**

Exudative retinal detachment in patients with multiple myeloma is exceedingly rare. Only two Cases are known to us. We successfully identified light-chain proteins in subretinal fluid, allowing for a more precise understanding of the pathogenesis of this complication.

**Case presentation:**

A 68-year-old patient presented with bilateral exudative retinal detachment. The visual impairment was reported one day after stem cell mobilization by granulocyte-colony stimulating factor (G-CSF) and the additional administration of Plerixafor. The symptoms began during stem cell apheresis. The patient underwent surgical treatment for both eyes through vitrectomy and silicone oil tamponade. Light-chain proteins were detected in the collected subretinal fluid through electrophoresis in one eye.

**Conclusions:**

We successfully identified light-chain proteins in subretinal fluid, allowing for a more precise understanding of the pathogenesis of this complication. The pathomechanism likely involves damage to the outer blood-retina barrier due to the deposition of light-chain proteins. Whether mobilization of bone marrow cells with Plerixafor led to a breakdown of the outer blood-retina barrier in these patients is a topic for discussion and has to be considered in the use of Plerixafor.

## Background

An exudative retinal detachment due to multiple myeloma is exceedingly rare. A patient with a complete bilateral exudative detachment of retinal layers is not known to us.

### Case presentation

A 68-year-old patient presented with bilateral visual impairment at our clinic. The patient reported a progressive decrease of vision in both eyes over the past two weeks. The symptoms first appeared during a stem cell apheresis. A kappa-type light chain myeloma has been known for 1.5 years (tumor stage 2 according to R-ISS). Initially, it was treated with four cycles of induction therapy following the Daratumumab-VTD protocol. A subsequent stem cell transplantation was planned. Subsequently, bone marrow mobilization was performed using by granulocyte-colony stimulating factor (G-CSF) and a day before the onset of bilateral visual impairment, additionally Plerixafor was administered. The stem cell apheresis took place the following day, and the symptoms described above began. Additionally, in the ophthalmological history, bilateral pseudophakia was noted.

**Fig. 1:**
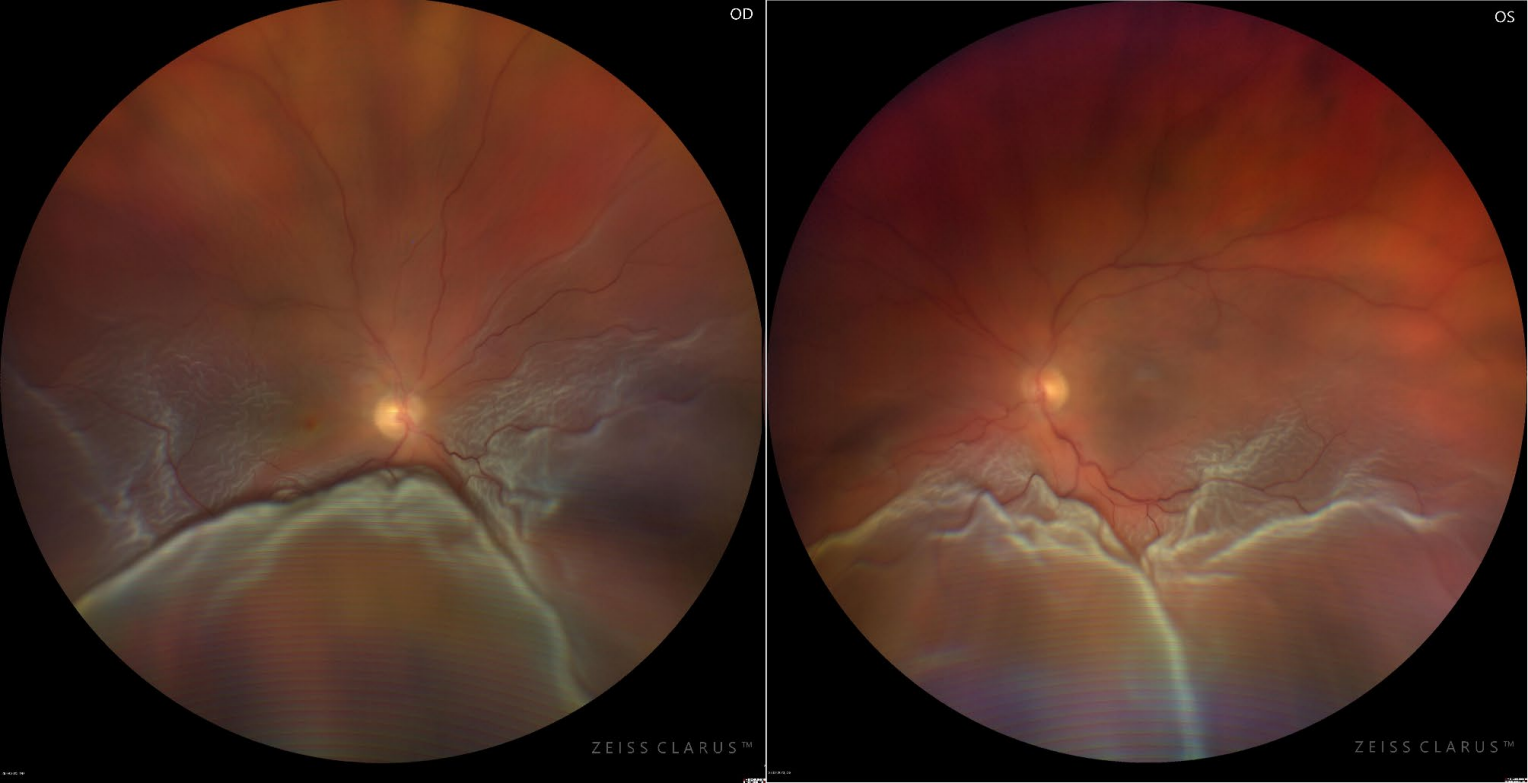
Photographs of the Retina. Left Image: Retina of the right eye. A total retinal detachment is evident with a highly bullous appearance inferiorly. Right Image: Retina of the left eye. A total retinal detachment is discernible with a highly bullous appearance inferiorly

The visual acuity was “hand movements” in the right eye and “counting fingers” in the left eye. The intraocular pressure was 10 mmHg in the right eye and 9 mmHg in the left eye (Goldman applanation tonometry). The anterior segment of both eyes was normal, no signs of cells. The Intraocular lenses were centered in both eyes. Fundoscopy revealed a total retinal detachment in both eyes. Superiorly, the retina was flatly detached, while inferiorly the retina showed a free floating highly detached retina (Fig. 1). Small bleedings were observed in some areas. A retinal tear could not be identified before surgery in either eye. A bilateral exudative retinal detachment was diagnosed.

### Therapeutic intervention

Because the underlying pathology of an exudative retinal detachment is a breakdown of the blood retina barrier, we recommended oral glucocorticoid therapy with prednisolone 60 mg once daily [1]. Also, we recommended, that the hematological treatment should be continued. In the follow-up examination 13 days later, no significant improvement was observed, leading to the recommendation of surgical intervention. Initially, the right eye underwent a pars plana vitrectomy (20-gauge) with collecting samples (vitreous body and subretinal fluid), infusion of perfluoro decalin (Fluoron F-Decalin), and an endotamponade of silicone oil (Bausch + Lomb Oxane 5700). The postoperative course was uneventful. On the first day a small circumscribed localized and shallow retinal detachment under silicone oil tamponade was noted. There was no sign of a retinal tear, so we concluded it was exudative. We decided to a wait and see approach.

Six days later, the left eye underwent the same surgical procedure. Postoperatively the retina was completely attached. The postoperative course was again uneventful. Three days later, the patient was discharged from stationary treatment. The visual acuity had improved to (uncorrected) 0,06 (Snellen) in the right eye and 0.16 (Snellen) in the left eye.

Taps from the vitreous as well as from the subretinal space from the left eye were obtained and analyzed for light-chains. The vitreous sample did not show evidence of involvement of the known light-chain myeloma or amyloid. However, electrophoresis successfully demonstrated the presence of free kappa and lambda light-chain proteins in the subretinal fluid of the left eye.

**Fig. 2:**
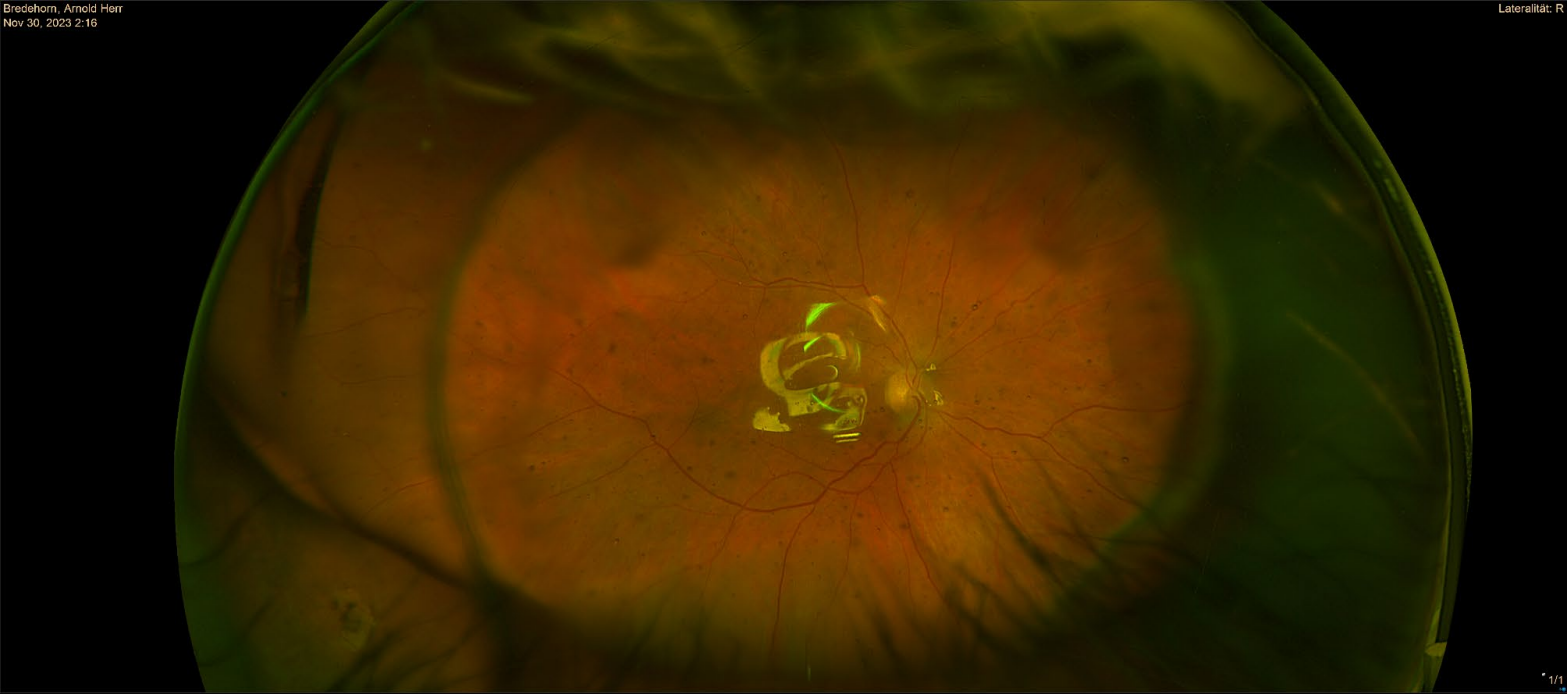
Postoperative Optos-Image from the right eye with silicon-oil endotamponade. The recurrant peripheral retinal detachment cannot be seen in this image

**Fig. 3:**
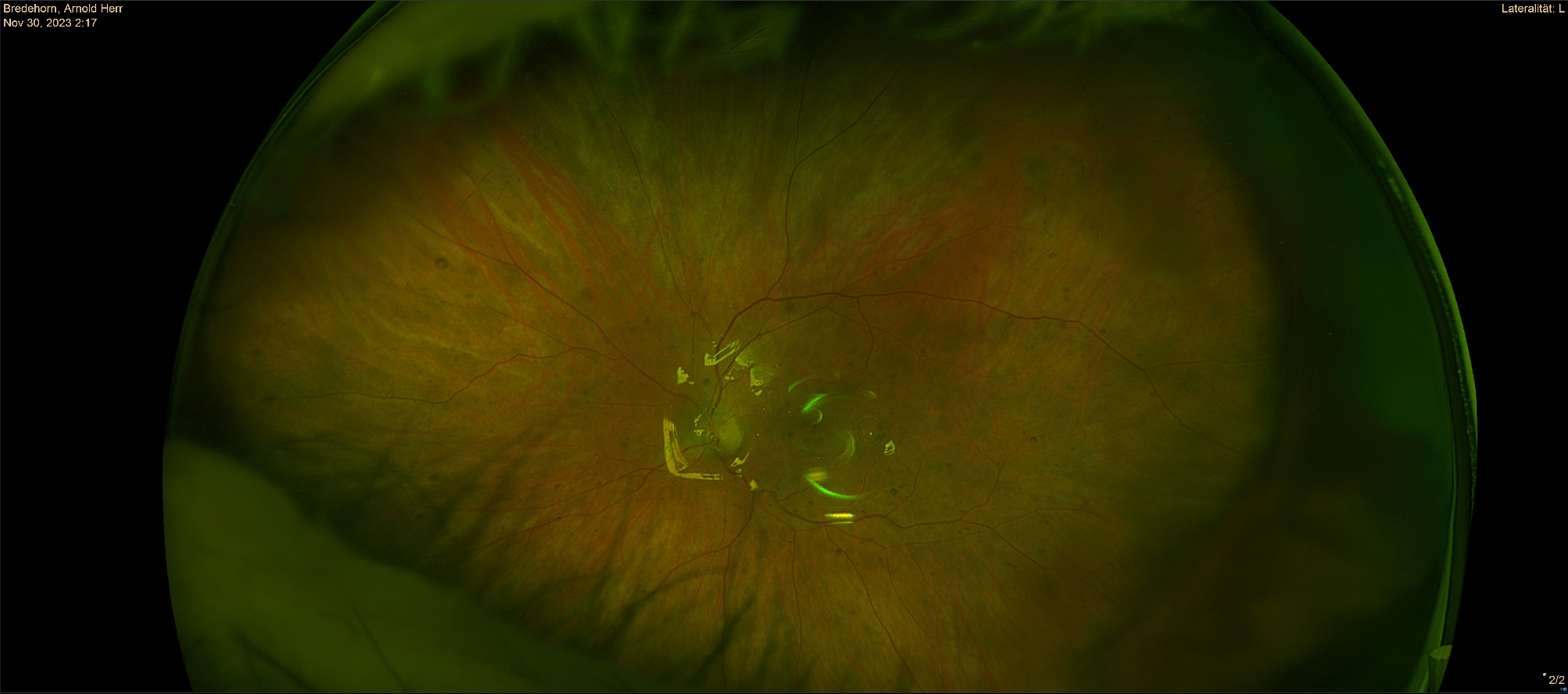
Postoperative Optos-Image from the left eye with silicon-oil endotamponade

Eight weeks after the inpatient stay, the patient presented for a follow-up examination at our clinic. A recurrent retinal detachment of the inferior periphery, first observed at the third postoperative day, was still observed in the right eye. The detachment showed no progress. The retinal detachment involved the inferior periphery, without involvement of the macula and without a sign of a retinal tear. *Because initially the glucocorticoid therapy showed no effect*,* we decided for a surgical approach.* (Fig. 2, 3). We performed a pars plana vitrectomy with exchange of the silicone oil and the placement of an encircling band on the right eye. Intraoperatively, no retinal hole was observed inferiorly as the cause of the retinal detachment. The postoperative course proceeded uneventfully. The patient was discharged after 3 days.

**Fig. 4:**
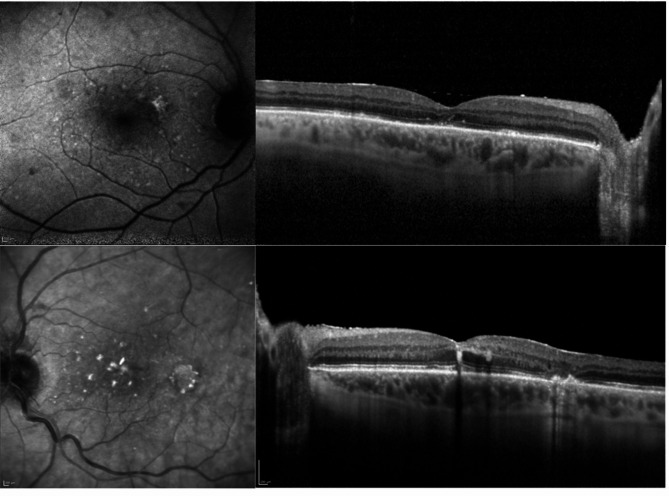
Upper Image: Postoperative Infrared-Image and OCT-Image from the right eye. It can be seen, that there some mild Alterations in the photoreceptor layer, but nearly normal retinal layers. Lower Image: Postoperative Infrared-Image and OCT-Image from the left eye. It can be seen, that there a some mild Alterations in the photoreceptor layer, but nearly normal retinal layers

## Discussion and conclusions

Ocular complications in patients with multiple myeloma are rare. These can be divided into two groups based on pathophysiology. The first group describes pathologies resulting from the infiltration or settling of plasma cells. The orbit is most involved in this group. Patients often present with double vision, visual impairment, and may clinically exhibit exophthalmos [2]. 

The second group describes pathologies arising from the hematologic changes triggered by multiple myeloma. Common findings in this group include crystalline corneal deposits, ciliary body cysts, and retinal changes [2]. These changes caused by the deposition of light-chain proteins and can manifest as vortex keratopathy or as diffuse stromal haze. Furthermore, case reports of non-infectious anterior and intermediate uveitis are known [3]. On the retina, changes ranging from mild tortuosity of blood vessels with hemorrhages to central retinal vein occlusion due to hyper viscosity syndrome are more common [3, 4]. 

An uncommon occurrence is the presence of subretinal fluid with detachment of the neurosensory retina [4]. In a case report from 2020, Wisely et al. [5]. described a 78-year-old patient with exudative retinal detachment inferiorly in the right eye, along with multiple retinal hemorrhages and cystoid macular edema with subretinal fluid in both eyes. At that time, the diagnosis of multiple myeloma had not yet been established [5]. 

Lam and Rodger [6] published a case report in 2014 about a 59-year-old woman with bilateral serous detachment of the central neurosensory retina in the macular region, venous stasis, and retinal hemorrhages. Grannis et al. [7]. also described a case with bilateral central sub foveal serous detachment of the neurosensory retina, which regressed under chemotherapy (Bortezomib, Dexamethasone) [7]. 

Rusu et al. [8]. reported a total of three patients with multiple myeloma and one patient with light-chain deposition disease. All patients exhibited central serous detachment of the neurosensory retina layer in the macular region [8]. 

And also Brody et al. [9] showed a patient with bilateral macular serous detachment.

However, all the mentioned cases showed a neurosensory serous detachment of the macula, not a peripheral exudative retinal detachment.

To the best of our knowledge, exudative detachment in the peripheral retinal area is described in only two cases [10]. A patient with a complete bilateral exudative detachment of retinal layers is not known to us. Nevertheless, the same pathomechanism could have led to an exudative retinal detachment in our case as well.

Daicker et al. [10] demonstrated through electron microscopy of a Bruch’s membrane that there were massive deposits of kappa light-chain proteins in a patient with multiple myeloma and Light-Chain Deposition Disease (LCDD) in an eye with exudative retinal detachment.

Pathophysiological, analogous to the basal membrane of the kidney, damage to the Bruch’s membrane could occur due to the deposition of light-chain proteins. This could lead to a breakdown of the outer blood-retina barrier, allowing serous fluid to accumulate between the neurosensory retina and the retinal pigment epithelium. Our detection of light-chain proteins in the subretinal fluid supports this hypothesis.

Plerixafor is a CXCR4 chemokine receptor antagonist approved for cell mobilization before stem cell apheresis in multiple myeloma. It could be speculated, whether a similar mechanism occurs in the break down in interphotoreceptor matrix. Also, it could be assumed that the therapy with Plerixafor for cell mobilization led to a significant flushing out of light-chain proteins and thus to a definitive breakdown of the outer blood-retina barrier. However, there is no description in literature or corresponding product information. Nevertheless, we have reported a relevant adverse event [11]. 

An analysis of the subretinal fluid for other serum proteins and the comparison of the ratio of light chain protein / total proteins in the subretinal fluid versus the same ratio of light chain protein / total proteins in the serum could answer whether the presence of light chain protein is the cause (active transport) or the consequence (passive transport) of the exudative detachment we observed. We suggest this analysis in future cases of this rare retinal complication in multiple myeloma patients. In summary, exudative retinal detachment in patients with multiple myeloma is a rare ocular complication. Whether this is related to Plerixafor remains unclear. We successfully identified light-chain proteins in subretinal fluid, allowing for a more precise understanding of the pathogenesis of the disease of multiple myeloma.

## Data Availability

The datasets used and/or analyzed during the current study are available from the corresponding author on reasonable request.

## References

[1] Amer R, Nalci H, Yalcindag N. Exudative retinal detachment. Surv Ophthalmol. 2017;62(6):723–69. 28506603 10.1016/j.survophthal.2017.05.001

[2] Orellana J, Friedman AH. Ocular manifestations of multiple myeloma, Waldenström’s macroglobulinemia and benign monoclonal gammopathy. Surv Ophthalmol. 1981;26(3):157–69. 6801795 10.1016/0039-6257(81)90065-5

[3] Singh RB, Singhal S, Sinha S, Cho J, Nguyen AX, Dhingra LS, et al. Ocular complications of plasma cell dyscrasias. Eur J Ophthalmol. 2023;33(5):1786–800. 36760117 10.1177/11206721231155974 PMC10472748

[4] Merz T, Marchesoni I, Caminiti G, Romanelli F. Efficacy of plasmapheresis as treatment for bilateral hyperviscosity syndrome related retinopathy in multiple myeloma. Eur J Ophthalmol. 2022;32(4):Np48–51. 33601903 10.1177/1120672121997069

[5] Wisely CE, Zhang W, Grewal DS. Multiple myeloma presenting as recalcitrant Macular Edema. J Vitreoretin Dis. 2020;4(3):248–52. 37007440 10.1177/2474126419880491 PMC9982255

[6] Lam LA, Rodger DC. Bilateral macular detachments, venous stasis retinopathy, and retinal hemorrhages as initial presentation of multiple myeloma: a case report. Retin Cases Brief Rep. 2014;8(4):240–4. 25372517 10.1097/ICB.0000000000000110

[7] Grannis CH, Dewan VN, Wang RC. Resolution of bilateral cystoid macular edema and subfoveal serous retinal detachments after treatment with bortezomib in a patient with smoldering multiple myeloma. Retin Cases Brief Rep. 2014;8(4):348–51. 25372546 10.1097/ICB.0000000000000067

[8] Rusu IM, Mrejen S, Engelbert M, Gallego-Pinazo R, Ober MD, Johnson MW, et al. Immunogammopathies and acquired vitelliform detachments: a report of four cases. Am J Ophthalmol. 2014;157(3):648–e571. 24321469 10.1016/j.ajo.2013.11.020

[9] Brody JM, Butrus SI, Ashraf MF, Rabinowitz AI, Whitmore PV. Multiple myeloma presenting with bilateral exudative macular detachments. Acta Ophthalmol Scand. 1995;73(1):81–2. 7627765 10.1111/j.1600-0420.1995.tb00019.x

[10] Daicker BC, Mihatsch MJ, Strøm EH, Fogazzi GB. Ocular pathology in light chain deposition disease. Eur J Ophthalmol. 1995;5(2):75–81. 7549446 10.1177/112067219500500202

[11] DiPersio JF, Stadtmauer EA, Nademanee A, Micallef IN, Stiff PJ, Kaufman JL, et al. Plerixafor and G-CSF versus placebo and G-CSF to mobilize hematopoietic stem cells for autologous stem cell transplantation in patients with multiple myeloma. Blood. 2009;113(23):5720–6. 19363221 10.1182/blood-2008-08-174946

